# Expanding the genetic landscape of Usher syndrome type IV caused by pathogenic *ARSG* variants

**DOI:** 10.1111/cge.14614

**Published:** 2024-08-28

**Authors:** Miriam Bauwens, Vincent De Man, Isabelle Audo, Irina Balikova, Wadih M. Zein, Vasily Smirnov, Sebastian Held, Sascha Vermeer, Elke Loos, Julie Jacob, Ingele Casteels, Julie Désir, Fanny Depasse, Stijn Van de Sompele, Mattias Van Heetvelde, Marieke De Bruyne, Camille Andrieu, Christel Condroyer, Aline Antonio, Robert Hufnagel, Ana Luísa Carvalho, João Pedro Marques, Christina Zeitz, Elfride De Baere, Markus Damme

**Affiliations:** ^1^ Center for Medical Genetics, Ghent University Hospital & Department of Biomolecular Medicine Ghent University Ghent Belgium; ^2^ Department of Ophthalmology University Hospital Leuven Leuven Belgium; ^3^ Sorbonne Université, INSERM, CNRS Institut de la Vision Paris France; ^4^ Centre Hospitalier National d'Ophtalmologie des Quinze‐Vingts Centre de Référence Maladies Rares REFERET and INSERM‐DGOS CIC 1423 Paris France; ^5^ Ophthalmic Genetics and Visual Function Branch NEI, NIH Bethesda Maryland USA; ^6^ Exploration de la Vision et Neuro‐Ophtalmologie CHU de Lille Lille France; ^7^ Christian‐Albrechts‐University Kiel Institute of Biochemistry Kiel Germany; ^8^ Center for Human Genetics University Hospitals Leuven Leuven Belgium; ^9^ Department of Otorhinolaryngology‐Head and Neck Surgery University Hospitals Leuven Leuven Belgium; ^10^ Department of Neurosciences, Research Group Experimental Oto‐Rhino‐Laryngology (ExpORL), KU Leuven University of Leuven Leuven Belgium; ^11^ Biomedical Sciences Group, Department of Neurosciences Research Group Ophthalmology Leuven Belgium; ^12^ Center for Medical Genetics Institut de Pathologie et de Génétique Gosselies Charleroi Belgium; ^13^ Department of Pediatric Ophthalmology Queen Fabiola Children's University Hospital (HUDERF) Brussels Belgium; ^14^ Pathology Department, Genetics Department, Center for Integrated Healthcare Research Kaiser Permanente Honolulu Hawaii USA; ^15^ Medical Genetics Unit Centro Hospitalar e Universitário de Coimbra (CHUC) Coimbra Portugal; ^16^ Clinical Academic Center of Coimbra (CACC) Coimbra Portugal; ^17^ University Clinic of Medical Genetics, Faculty of Medicine University of Coimbra (FMUC) Coimbra Portugal; ^18^ Ophthalmology Unit Centro Hospitalar e Universitário de Coimbra (CHUC) Coimbra Portugal; ^19^ University Clinic of Ophthalmology, Faculty of Medicine University of Coimbra (FMUC) Coimbra Portugal; ^20^ Institute for Clinical and Biomedical Research (iCBR), Faculty of Medicine University of Coimbra (FMUC) Coimbra Portugal

**Keywords:** ARSG, canine variant p.(Arg99His), lysosomal sulfatase, retinitis pigmentosa (RP), rod‐cone dystrophy (RCD), sensorineural hearing loss (SNHL), Usher syndrome (USH)

## Abstract

Usher syndrome (USH) is the most common cause of deafblindness. USH is autosomal recessively inherited and characterized by rod‐cone dystrophy or retinitis pigmentosa (RP), often accompanied by sensorineural hearing loss. Variants in >15 genes have been identified as causative for clinically and genetically distinct subtypes. Among the ultra‐rare and recently discovered genes is *ARSG*, coding for the lysosomal sulfatase Arylsulfatase G. This subtype was assigned as “USH IV” with a late onset of RP and usually late‐onset progressive SNHL without vestibular involvement. Here, we describe nine new subjects and the clinical description of four cases with the USH IV phenotype bearing seven novel and two known pathogenic variants. Functional experiments indicated the complete loss of sulfatase enzymatic activity upon ectopic expression of mutated *ARSG* cDNA. Interestingly, we identified a homozygous missense variant, p.(Arg99His), previously described in dogs with neuronal ceroid lipofuscinosis. Our study expands the genetic landscape of *ARSG*‐USH IV and the number of known subjects by more than 30%. These findings highlight that USH IV likely has been underdiagnosed and emphasize the need to test molecularly unresolved subjects with deafblindness syndrome. Finally, testing of *ARSG* should be considered for the genetic work‐up of apparent isolated inherited retinal diseases.

## Graphical abstract

Mutations in *ARSG* are a very rare cause of Usher disease, assigned as “Usher type IV.” We identified 13 new subjects with mutations in *ARSG*, provide a clinical description of the subjects, and characterized the newly identified variants.

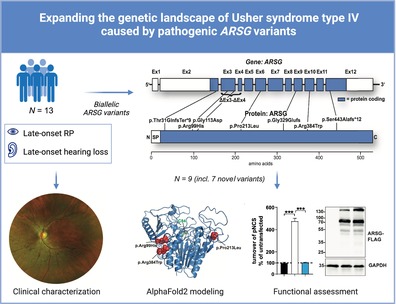

## 1 INTRODUCTION

Usher syndrome (USH) is the most common cause of deafblindness in humans, with an overall global prevalence of about 1/10 000. This autosomal recessively inherited disease is typically characterized by sensorineural hearing loss (SNHL) and inherited retinal disease (IRD), specifically rod‐cone dystrophy (RCD) or retinitis pigmentosa (RP).^1^ Its clinical presentation is heterogeneous with three major subtypes, USH1, 2, and 3, depending on the onset and severity of hearing loss (HL), vestibular dysfunction, and the age of onset of IRD. Nine USH genes have been implicated in these subtypes,^1^ encoding proteins playing key roles in auditory sensory cell development, function, and photoreceptor maintenance.^1^ The general term “USH” has also been used for diseases associated with variants in genes with less clear function in the retina and auditory sensory system, with little or no evidence of interactions with any classical USH genes. With the Usher classification being purely based on the clinical presentation of IRD and SNH, these subtypes are assigned as “atypical USH.”

A more recently identified atypical USH gene is *ARSG*, encoding the lysosomal sulfatase Arylsulfatase G (ARSG).^2^ We have previously shown that ARSG cleaves sulfate groups from 3‐*O* sulfated N‐acetylated glucosamine in heparan sulfate (HS), with knockout (KO) *Arsg* mice accumulating 3‐*O* sulfated HS in lysosomes.^3^ Accumulation of HS can be observed in the central nervous system and peripheral organs, with KO mice generally presenting the typical signs of a lysosomal storage disorder (LSD).^3^, ^4^ These mice show signs of early retina degeneration, with ataxia at later stages.^5^ A natural model with a homozygous missense variant in *ARSG* p.(Arg99His) was found in dogs, displaying LSD and severe neurological symptoms similar to the KO mice.^6^

In humans, a homozygous founder variant in *ARSG*, c.133G>T p.(Asp45Tyr), was identified in families with IRD and SNHL. This new subtype was tentatively assigned as “USH IV.”^2^ The distinctive retinal phenotype was characterized by ring‐shaped atrophy along the arcades engirdling the fovea, resulting in ring scotoma. Both vision and hearing loss appeared around the age of 40 years.^2^ After the initial identification of these cases, additional subjects from different ethnicities were identified with coding loss‐of‐function variants.^7^, ^8^, ^9^, ^10^, ^11^, ^12^, ^13^ To date, 22 cases from different ethnicities with loss‐of‐function variants have been described, highlighting this subtype's rarity.^7^, ^8^, ^9^, ^10^, ^11^, ^12^, ^13^ All *ARSG* cases were diagnosed with RP, and HL is present in all reported subjects, one excepted.^8^ With one exception of a patient with mild cerebellar degeneration, no neurological symptoms have been reported.^12^ This is remarkable, given that loss of ARSG activity in animal models leads to typical LSD.^3^, ^6^ The apparent animal‐human discrepancy has yet to be explained.

Here, we present nine new cases and the clinical characterization of four known subjects^13^ with biallelic variants in *ARSG*, expanding the number of USH IV subjects from 22 to 31. We broadened the genetic spectrum of *ARSG* and validated the novel variants by assessing enzymatic activity. Moreover, we identified one case with typical USH IV who did not display any neurological symptoms despite being homozygous for the “canine” p.(Arg99His) LSD variant.^6^

## 2 MATERIALS AND METHODS

### 2.1 Ethics/consent

This study was performed in line with the principles of the Declaration of Helsinki. Approval was granted by all involved centers and universities. Informed consent was obtained from all individual participants included in the study.

### 2.2 Recruitment and clinical evaluation of subjects

The nine newly described subjects and four reported ones described in this study were recruited from five centers (Center for Medical Genetics, Ghent University, Ghent, Belgium; University Hospital Leuven, Leuven, Belgium; Reference Center for Rare Diseases RefeRet at Centre Hospitalier National d'Ophtalmologie des Quinze‐Vingts, Paris, France; Ophthalmic Genetics and Visual Function Branch, National Eye Institute, National Institutes of Health, Bethesda, USA; CHUC, Coimbra, Portugal). EDTA blood samples were obtained from the probands and available family members. DNA was isolated from peripheral blood lymphocytes by standard procedures.

See Supplemental Methods for a detailed description of the clinical examinations of the subjects in the different centers.

### 2.3 Whole exome sequencing, targeted next‐generation sequencing, and Sanger sequencing

Affected subjects 1 and 2 underwent whole exome sequencing (WES) using SureSelectXT Low Input Human All Exon V7 enrichment (Agilent Technologies) and NovaSeq 6000 sequencing (Illumina). Data analysis was executed using pipelines developed in‐house, and was limited to 290 genes associated with IRD present in the RetNet gene panel (version 5, www.cmgg.be). Affected subject 3 underwent genetic testing by WES with a commercial IRD panel (Blueprint Genetics retinal dystrophy panel). Affected subjects 4, 5, 6, and 8 underwent targeted NGS, as previously described.^14^ Four subjects (subjects 9–12) were genetically identified and reported before (CHUC, Coimbra, Portugal).^13^ Subjects 9 and 10 are brother and sister. The proband (brother) underwent WES‐based panel testing of 13 genes associated with USH, including copy number variant (CNV) analysis—https://www.unilabs.pt/en/exam/18/3742; familial variant testing was used to diagnose the sister. Subject 12 also underwent a WES‐based panel testing of 13 genes associated with USH, including CNV analysis. Subject 12 underwent a WES‐based panel testing of 302 genes associated with IRD, including CNV analysis (https://www.unilabs.pt/en/exam/18/3918), and Subject 13 underwent WES including CNV analysis (https://www.unilabs.pt/en/exam/18/9/2756). Segregation analysis in available family members (parents and/or siblings) was performed for subjects 1, 2, and 5 via PCR and Sanger sequencing. Selected variants were classified based on the ACMG and ACGS guidelines with adaptations^15^, ^16^, ^17^, ^18^ using an in‐house developed classification tool.

### 2.4 Quantitative reverse transcription‐polymerase chain reaction (RT‐PCR)

Quantitative RT‐PCR experiments were performed with oligonucleotides partially located in exon 2 (forward: 5′‐CAGTGGGA AAACAAGA‐3′ and reverse: 5′‐AGACTCACCTCATTCCCTCC‐3′), exon 3 (forward: 5′‐CCCTGCAGGTTTGTGG ATTT‐3′ and reverse: 5′‐AAGTTGCGTGTGACTCCATT‐3′), exon 4 (forward: 5′‐AGTGTGTGATCAGCTCCTCA‐3′), exon 5 (forward: 5′‐AAAGGCAAGCACATGGAGTC‐3′ and reverse: 5′‐GGTGGTTGTAGCCTGGAGTA‐3′) of *ARSG* using the Power SYBR‐Green PCR Master Mix kit (Applied Biosystems, Life Technologies) at 60°C annealing temperature according to the manufacturer's protocol.

### 2.5 Molecular biology and biochemical methods

Immunoblot and sulfatase activity assays were performed as described previously.^7^ The cDNA expression vectors coding for C‐terminally 3xFLAG‐tagged wildtype and mutated *ARSG* were generated by PCR and cloning into the pcDNA4/TO‐C‐3xFlag vector with *EcoR*I and *Xho*I followed by transfection of HT1080 cells and selection of stable cell lines by Zeocin over 2–3 weeks. The variants were introduced by site‐directed mutagenesis (QuikChange II site‐directed mutagenesis kit, Agilent, Santa Clara, CA, USA) according to the manufacturer's protocol and as described before^7^ with the following primers for mutagenesis: c.1150C>T, p.(Arg384Trp), forward: 5′‐TCAAAGCGCCATCCTTGAGGTAAGCTGGCC‐3′; reverse: 5′‐GGCCAGCTTACCTCAAGGATGGCGCTTTGA‐3′; c.296G>A, p.(Arg99His), forward: 5′‐GTGTGACTCCATTGTGAAGGCCAAGCCGG‐3′; reverse: 5′‐CCGGCTTGGCCTTCACAATGGAGTCACAC‐3′; c.638C>T, p.(Pro213Leu) forward: 5′‐GCTGCTCAAGTTCACCAGCTGCTCCACAATGTT‐3′, reverse: 5′‐AACATTGTGGAGCAGCTGGTGAACTTGAGCAGC‐3′. All constructs were sequence‐validated by Sanger sequencing. HT1080 cells were transfected as described previously.^7^, ^10^ HT1080 cells were treated with 600 μg/mL Zeocin (Invivogen) 3 days after transfection, and stable cell lines were selected by passaging in a Zeocin‐containing medium after at least two passages. Immunoblotting of lysates from the stably transfected cells was performed according to standard procedures by semi‐dry blotting on nitrocellulose membrane and using antibodies against FLAG‐tag (clone M2; Sigma‐Aldrich) and GAPDH as a loading control (SC‐335; Santa Cruz Biotechnology). Lysates were prepared by harvesting the cells by scraping the cells from 10‐cm dishes in PBS containing complete protease inhibitors (Sigma‐Aldrich), washing the cells with PBS, and lysis in 50 mM Tris–HCl pH 7.4, 150 mM NaCl, 1% (w/v) Triton X‐100, 0.1% (w/v) SDS + Complete protease‐inhibitor cocktail followed by ultrasonification for 20 s. Lysates were cleared by centrifugation for 10 min at 13.000 *g*, and the supernatant was used for immunoblotting and activity assays. The total cellular sulfatase activity was measured photometrically by the turnover of p‐nitrocatechol sulfate (pNCS) (Sigma‐Aldrich) from protein lysates of stable HT1080 cell lines with the wildtype *ARSG* construct or the construct harboring the indicated *ARSG* variants as described.^7^, ^10^

## 3 RESULTS

### 3.1 Clinical evaluation of subjects

Table 1 summarizes the major findings of all 13 investigated subjects.

**TABLE 1:** Clinical data and findings of the subjects described in this study.

ID	Sex	Age of visual symptom onset (y)	Age of 1st visit (y)	Ocular symptoms	Cataract	Macular findings	ffERG	SNHL onset (y)	Vestibular symptoms	MRI
Subject 1	M	65	65	Night vision disturbances and reduced peripheral vision	No	Perifoveal loss of the outer retinal layers	Rod‐cone dysfunction	47	None	Normal at the age of 47
Subject 2	F	25	n.a.	Night vision difficulties and visual field constriction	n.a.	Preserved outer retinal layers with cystoid macular edema	Rod‐cone dysfunction	n.a.	n.a.	n.a.
Subject 3	F	42	57	Night vision and visual field difficulties	Trace nuclear sclerosis at 67 y	Perifoveal progressive loss of outer retinal layers	Rod‐cone dysfunction	45	None	Mild cerebral and cerebellar atrophy. White matter lesions suggestive of small vessel vasculopathy
Subject 4	M	50s	53	Night vision disturbances	Subtle posterior subcapsular cataracts	Preserved outer retina with cystoid maculopathy	Rod‐cone dysfunction	None when last seen at 54	None	NP
Subject 5	F	18	59	Night vision disturbances and visual field constriction	Subtle posterior subcapsular cataracts	Preserved outer retina with cystoid maculopathy	Rod‐cone dysfunction	20	None	NP
Subject 6	F	58	63	Night vision disturbances	Subtle posterior subcapsular cataracts	Preserved outer retina with cystoid maculopathy	Rod‐cone dysfunction	58	None	NP
Subject 7	M	40	74	Night vision disturbances, then photophobia	Subtle posterior subcapsular and nuclear cataracts	Macular atrophy with a narrowed area of foveal sparing	Rod‐cone dysfunction	50	None	NP
Subject 8	M	55	n.a.	Night blindness and photophobia	Subtle posterior subcapsular	Cystoid maculopathy in the left eye with well preserved outer retina	Rod‐cone dysfunction	n.a.	None	NP
Subject 9	M	45	68	Decreased visual acuity, night vision disturbances and visual field constriction	Cataract surgery OU	Macular atrophy	Rod‐cone dysfunction	42	None	NP
Subject 10	F	50	72	Night vision disturbances and visual field constriction	Cataract surgery OU	Preserved outer retina	Rod‐cone dysfunction	30	None	NP
Subject 11	M	22	54	Decreased visual acuity, night vision disturbances and visual field constriction	Cataract surgery OD; cortical and nuclear cataract OS	Macular atrophy	Rod‐cone dysfunction	40	None	NP
Subject 12	F	52	55	Night vision disturbances and visual field constriction	No	Perifoveal progressive loss of outer retinal layers	Rod‐cone dysfunction	48	None	NP
Subject 13	F	34	36	Decreased visual acuity, night vision disturbances and photophobia	No	Macular atrophy	Rod‐cone dysfunction	48	None	NP

Abbreviations: F, female; ffERG, full‐field ERG; M, male; MRI, Magnetic Resonance Imaging; n.a., information not available; NP, not performed; OD, *oculus dexter* (right eye); OS, *oculus sinister* (left eye); OU, *oculus uterque* (both eyes); SNHL, sensorineural hearing loss; y, year.

**FIGURE 1:**
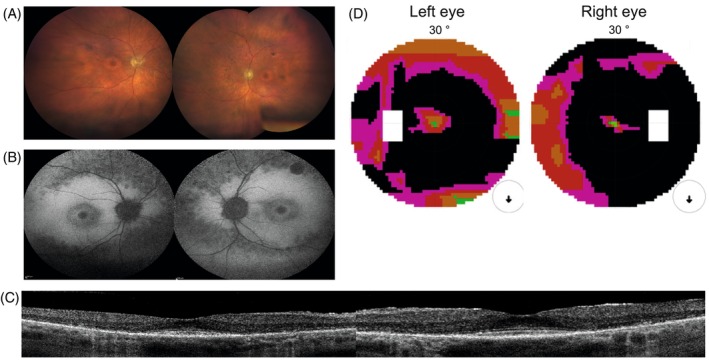
Clinical findings from subject 1: (A) fundus photography. Midperipheral is a zone of atrophy resembling a bull's eye maculopathy. The retinal vasculature is mildly attenuated. Nasally and superior of the optic disc intraretinal pigmentation as bone spicules are present. The retinal vasculature was mildly attenuated, and intraretinal pigmentation as bone spicules could be observed, mostly located nasally and superior to the optic disc. The peripheral retina was relatively preserved. (B) FAF. A normal foveal autofluorescence, a hyper‐autofluorescent perifoveal border, two concentric zones with consecutive one hypo‐autofluorescent and a larger hyper‐autofluorescent area, a confluent pattern of hypo‐autofluorescence around the vascular arcades and a normal autofluorescent peripheral retina. FAF imaging showed a normal foveal autofluorescence demonstrating the foveal sparing in this subject, a hyper‐autofluorescent perifoveal border, two concentric zones with consecutive one hypo‐autofluorescent and a larger hyper‐autofluorescent area, a confluent pattern of hypo‐autofluorescence around the vascular arcades and a normal autofluorescent peripheral retina. (C) On OCT, there was a thinned retina due to the loss of the outer retinal layers in the perifoveal area without cystoid spaces. Full‐field electroretinography (ERG) showed combined scotopic and photopic dysfunction, which was more pronounced for the scotopic responses, indicating rod‐cone dysfunction. (D) A ring scotoma between 10° and 20°. Central visual field testing showed a ring scotoma between 10° and 20°. Goldmann's peripheral visual field detected only a mild reduction of the peripheral borders.

*Subject 1* of Belgian descent presented at 65 years of age with signs of RP. Best corrected visual acuity (BCVA) is 20/20 in the right and 20/22 in the left eye. Problems with color vision were noted from 30 years old, supported by an abnormal Ishihara test. A detailed description of fundus photography, fundus autofluorescence (FAF), optical coherence tomography (OCT), and central visual field testing can be found in Figure 1A–D. The visual fields showed a minor reduction. He was diagnosed with down‐sloping SNHL (30 dB HL at the lower frequencies, down‐sloping until 70–80 dB HL at 8000 Hz) at the age of 47 years and had bilateral hearing aids. At the age of 50, an ENT specialist was consulted for episodes of dizziness but no vestibular impairment was found. A brain MRI, including the cerebellopontine angle, was normal and his mother was known to have hearing difficulties (not further specified).

*Subject 2* was initially diagnosed with RP at 28 years of age. At the age of 25, decreased night vision was already present, followed by complaints of a loss of central vision. The BCVA was 20/40 in each eye. Fundus examination showed typical bone spicule pigmentary changes (Supplemental Figure 2). The visual fields showed a peripheral reduction more pronounced in the upper part in each eye. Spectral‐domain (SD‐OCT) showed preserved outer retinal layers at the macula with cystoid macular edema in each eye. Full field ERG showed a pattern of RCD, with reduced b wave amplitude in scotopic conditions. The photopic response was almost preserved. FAF showed a ring of parafoveal hyper‐autofluorescence, a central spot of hyper‐autofluorescence, and more peripherical hypo‐fluorescence changes. No mention was made of HL.

*Subject 3* was referred for IRD at 57 years of age. Night blindness and visual field defects were first noted in her early forties. The BCVA at that time was 20/20 in each eye. Color vision was normal. (Micro)perimetry revealed a ring scotoma with preservation of the central 10°. Fundus exam showed well‐preserved maculae. FAF showed a ring of parafoveal hyper‐autofluorescence and hypo‐autofluorescent changes (Supplemental Figure 1). OCT showed ellipsoid zone loss (EZ) corresponding to the area of chorioretinal atrophy. ERG responses were reduced in amplitude (mainly scotopic). Multifocal ERG showed a mild reduction. Audiologic history showed bilateral tinnitus at 35, followed by a gradual subjective HL. Bilateral SNHL (high‐frequency) was diagnosed at 45. Balance was normal, confirmed by vestibular testing. A return visit 12 years later showed severe HL, an expanded ring scotoma, and further loss of EZ. Mild cerebral and cerebellar atrophy was evident on MRI, and white matter lesions are suggestive of small vessel vasculopathy.

*Subject 4* is a male patient of Portuguese descent. He was referred at age 53 for visual field constriction. He has reported night blindness for several years and denied any HL. BCVA was 20/40 in the right eye 20/100 in the left eye. Fundus examination revealed typical signs of RCD. Short‐wavelength autofluorescence imaging shows a patchy loss of autofluorescence outside the vascular arcades, a ring of increased autofluorescence around the macula, and a relatively normal autofluorescence at the fovea. SD‐OCT showed well‐preserved outer retinal layers at the macula with cystoid maculopathy only on the left. The patient was lost from follow‐up.

**FIGURE 2:**
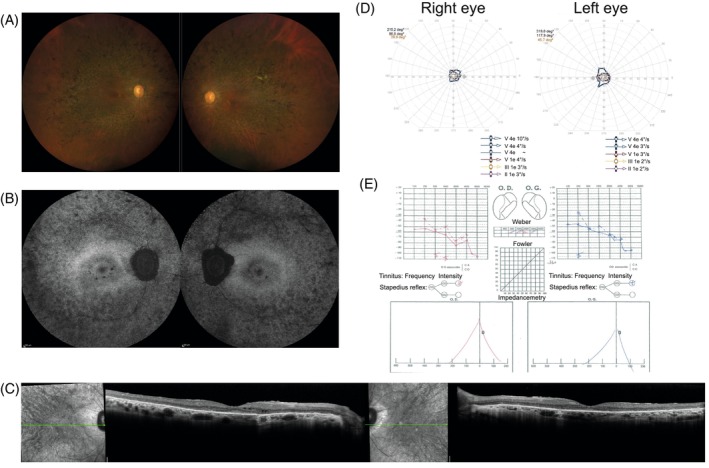
Clinical findings from subject 5: (A) fundus photographs reveal typical signs of RCD/RP, including a waxy pallor of the optic discs, narrowed retinal vessels, and pigmentary changes outside the vascular arcardes; (B) short‐wavelength fundus autofluorescence imaging shows a patchy loss of autofluorescence in the periphery, a normal autofluorescence appearance at the fovea and some patches of loss of autofluorescence in the parafoveal region; (C) horizontal SD‐OCT scans show a loss of hyperreflective bands and a thinning of the outer nuclear layer, outside the foveal region, with a well preserved outer retina at the fovea and some intraretinal cysts parafoveal on the right eye; (D) kinetic visual fields are severely constricted for both eyes; (E) audiometry shows a loss of sensitivity for the higher frequencies for both ears.

*Subject 5* is the 7th female child of a non‐consanguineous Portuguese couple with an older sister also affected. She was diagnosed with RCD at 18 years due to night blindness. On the last examination, she was 59 years old with a BCVA of 20/40 in the right and 20/50 in the left eye. Visual fields were bilaterally constricted, reduced to 10°. Multimodal retinal imaging was typical of RCD with a relative foveal sparing (Figure 2A–D). At age 20, audiometry revealed unilateral HL, progressing towards bilateralism with midfrequency SNHL (Figure 2E). Her 4‐year older sister, *subject 6*, had been diagnosed only at age 58 with milder RCD, although similarly associated with progressive HL and cystoid maculopathy. At age 63, she maintains a 20/25 BCVA in both eyes with a kinetic visual field reduced to 20 central degrees bilaterally.

*Subject 7* was first seen at 74. He is the 5th child of a consanguineous Tunisian couple. He reported night blindness since his early 40s with secondary photophobia and progressive HL in his 50s. At presentation, BCVA was 20/80 for the right eye and 20/63 for the left. Visual fields only revealed the perception of the stimuli inferiorly. Multimodal imaging showed diffused pigmentary changes of the posterior pole and peripheral retina with only a narrowed area of foveal sparing.

*Subject 8* is a 55‐year‐old male French subject with no family history of visual impairment. At presentation, BCVA was 20/20 OD. Fundus exam revealed typical signs of RP, pigment migration in the periphery, a macular ring of increased autofluorescence, and loss of autofluorescence in the periphery. The OCT showed a small cyst on the right, large cysts on the left, and preserved outer retinal bands at the macula. The full‐field ERG showed non‐detectable responses under scotopic conditions and residual photopic responses, in keeping with generalized rod‐cone dysfunction. Kinetic perimetry of the subject revealed an annular scotoma in both eyes. He denied HL; audiometry was not performed.

Five subjects (four families, *subjects 9–13*) with *ARSG*‐related Usher syndrome were identified using the IRD‐PT registry^13^, ^19^ with consanguinity in three families. All patients were diagnosed with USH after 50 years of age, even though the first visual symptoms started in early adulthood in patient 11, adulthood in patients 9 and 12, and after 51 years of age in patients 10 and 13. Great phenotypic heterogeneity was observed by multimodal imaging (Supplemental Figures 3 and 4). Preserved central visual acuity (≥20/32) was seen in patients 10 (age 73) and 12 (age 56), both with a parafoveal ring of hyper‐autofluorescence on fundus autofluorescence. On the other hand, patients 7 (age 71), 11 (age 63), and 13 (age 51) present macular atrophy and BCVA < 20/400 in at least one eye.

### 3.2 Identification of novel and known loss‐of‐function *ARSG* variants

**FIGURE 3:**
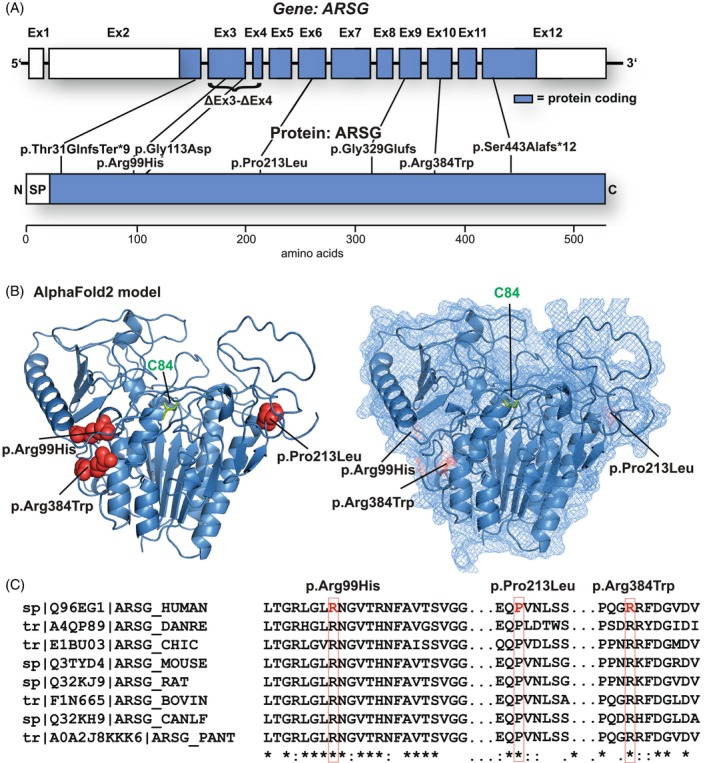
Mutational spectrum of the USH type IV cohort: (A) distribution of the identified variants within both the *ARSG* gene (upper panel, subject exons are depicted) and the primary amino acid sequence of the ARSG protein. (B) Distribution of the novel missense variants in AlphaFold2 (https://alphafold.ebi.ac.uk/entry/Q96EG1; left: Secondary structure elements shown as a cartoon; right: The protein surface is additionally shown transparently). The mutated amino acids are highlighted in red and depicted as spheres. The active‐site cysteine C84 is highlighted in green. (C) Multi‐species alignment of the protein sequence of ARSG for each identified missense variant and the surrounding amino acids.

We identified nine pathogenic *ARSG* variants in the nine index subjects by WES and targeted NGS testing (Table 2, Figure 3A, Supp. Table 1).

**TABLE 2:** Table with identified *ARSG* variants including c.nomenclature (according to transcript NM_001267727.2), corresponding p.nomenclature, type of mutation, zygosity, and available published data/reference for each of the identified variants.

ID	Variant (cDNA) (NM_001267727.2) (ENST00000448504.6)	Mutation type	Variant (protein)	Zygosity	Reference
Subject 1	c.91del	Frameshift	p.(Thr31Glnfs*9)	Homozygous	This study
Subject 2	c.986del	Frameshift	p.(Gly329Glufs*35)	Heterozygous	This study
	c.338G>A	Missense	p.Gly113Asp	Heterozygous	Peter et al.^10^
Subject 3	c.638C>T	Missense	p.Pro213Leu	Homozygous	This study
Subject 4	c.1150C>T	Missense	p.Arg384Trp	Heterozygous	This study
	c.219_454del	Frameshift	p.(Val75*)	Heterozygous	This study
Subject 5	c.1150C>T	Missense	p.Arg384Trp	Heterozygous	This study
	c.338G>A	Missense	p.Gly113Asp	Heterozygous	Peter et al.^10^
Subject 6	c.1150C>T	Missense	p.Arg384Trp	Heterozygous	This study
	c.338G>A	Missense	p.Gly113Asp	Heterozygous	Peter et al.^10^
Subject 7	c.296G>A	Missense	p.Arg99His	Homozygous	This study
Subject 8	c.1303+5G>T	Splice	p.?	Heterozygous	This study
	c.296G>A	Missense	p.Arg99His	Heterozygous	This study
Subject 9	c.1326del	Frameshift	p.(Ser443Alafs*12)	Homozygous	Felde et al.
Subject 10	c.1326del	Frameshift	p.(Ser443Alafs*12)	Homozygous	Felde et al.
Subject 11	c.1326del	Frameshift	p.(Ser443Alafs*12)	Homozygous	Felde et al.
Subject 12	c.338G>A	Missense	p.Gly113Asp	Homozygous	Peter et al.^10^
Subject 13	c.1150C>T	Missense	p.Arg384Trp	Homozygous	This study

In *subject 1*, WES revealed a novel homozygous frameshift variant c.91del, p.(Thr31Glnfs*9), predicted to lead to a premature stop codon inducing the transcript to undergo nonsense‐mediated decay (NMD), thus corresponding to a complete loss‐of‐function allele. Segregation analysis revealed heterozygous carriership of c.91del in his unaffected children.

*Subject 2* is a heterozygous carrier of the previously reported c.338G>A, p.(Gly113Asp) variant,^10^ predicted to be pathogenic (Supp. Table 1). Functional assays performed by us^10^ indicated that the mutant ARSG containing this missense change displays a complete loss of enzymatic activity. In addition, a novel frameshift variant c.986del p.(Gly329Glufs*35), predicted to initiate NMD, was identified. Segregation analysis revealed both variants to be in *trans*, with the frameshift variant being paternal.

Initial IRD panel testing for *subject 3* revealed one pathogenic variant in *ABCA4* and a variant of uncertain significance in the *CTNNA1* gene. WES revealed the ultra‐rare homozygous *ARSG* c.638C>T p.(Pro213Leu) variant, predicted to be pathogenic by in silico prediction tools (Supp. Table 1).

In *subject 4*, a novel and rare heterozygous missense variant c.1150C>T, p.(Arg384Trp) was identified and is predicted to be pathogenic by multiple in silico prediction tools (Supp. Table 1). In addition to this change, a heterozygous genomic deletion of exons 3 and 4 was identified and validated using qPCR. This copy number change is out‐of‐frame and predicted to lead to a premature stop codon p.(Val75*).

*Subject 5* carries the same heterozygous missense variant c.1150C>T, p.(Arg384Trp) as subject 4, in combination with a previously reported missense variant c.338G>A, p.(Gly113Asp) also identified in subject 2.^10^ The affected sister of this subject (*subject 6*) carries the same variants.

In *subject 7*, a novel homozygous missense variant c.296G>A, p.(Arg99His) was detected. In silico tools are suggestive of a pathogenic effect (Supp. Table 1). Notably, the mutated Arg99 is highly conserved, and this variant has already been described in dogs presenting with LSD and ataxia.^6^ This variant was also observed heterozygously in *subject 8* in conjunction with an ultra‐rare splice variant c.1303+5G>T, predicted to strongly impact splicing according to SpliceAI and in silico splice prediction tools (Supp. Table 1), which could result in an out‐of‐frame skipping of exon 11, predicted to lead to a premature stop codon; p.(Leu406Valfs*20).

A targeted NGS panel for Usher syndrome (WES‐based NGS panel of 13 genes, including CNV analysis) was used to diagnose the three affected subjects (*subjects 9–11*) from the IRD‐PT registry, revealing the previously reported c.1326del p.(Ser443Alafs*12) variant^7^ in a homozygous manner in affected subjects from two different families.

Lastly, *subject 12* underwent a WES‐based NGS Panel of 302 genes, including CNV analysis, while *subject 13* underwent WES, including CNV analysis. The previously described variant c.338G>A, p.(Gly113Asp), which was also identified in subject 2, was found in a homozygous manner in subject 12, while novel variant c.1150C>T, p.(Arg384Trp), also present in subjects 4, 5, and 6, all from Portuguese descent was identified homozygously in subject 13.

### 3.3 3D‐modeling of ARSG and localization of variants in the modeled structure

As an experimental structure of ARSG has not yet been published, we used AlphaFold2 to localize the novel missense variants in the predicted ARSG structure (Figure 3C). Arg384 is a surface‐exposed amino acid in an area with little secondary structure close to an alpha helix. The arginine to tryptophan with its bulky side chain, however, likely affects the overall structure and fold. Interestingly, Arg99 is very close to Arg384. Again, the p.(Arg99His) variant likely leads to changes in the overall protein structure. The proline, which is changed to leucine in position 213 in subject 4, is a well‐conserved residue in a core turn in the α‐helices of the enzyme and appears to act with other helices, supporting the likely deleterious effect of this substitution.

Finally, we compared the amino acid sequence of different ARSG orthologs for these same positions (Figure 3C) (Arg99, Pro213, and Arg384), amino acids were identical between all compared species, suggesting a high degree of conservation and critical functions in the protein structure/enzymatic activity.

### 3.4 Functional validation of identified variants

**FIGURE 4:**
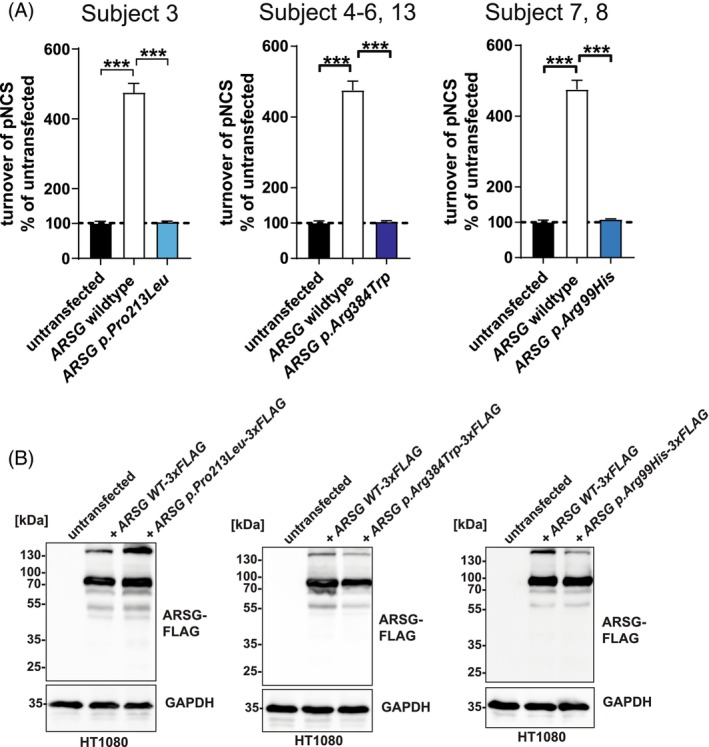
Functional analysis of missense variants in *ARSG* found in this study. (A) Total sulfatase activity of cell lysates from untransfected cells, or cells transfected with WT ARSG and mutated variants, against the artificial substrate pNCS, represented as the percentage of turnover in untransfected cells. *N* = 3 biological replicates and three technical replicates. Unpaired, two‐tailed *t*‐test, mean ± SEM of the biological replicates are shown. (B) Immunoblot analysis of untransfected or stably transfected HT1080 cells, with plasmids coding for 3xFLAG tagged ARSG (WT), along with its form carrying the pathogenic variants (p.Pro213Leu, p.Arg384Trp, and p.Arg99His). Antibodies are against FLAG; GAPDH was used as a loading control.

We identified seven novel variants and two previously described variants (p.(Gly113Asp) and p.(Ser443Alafs*12)).^10^, ^20^ Three of these variants are frameshifting variants: p.(Thr31Glnfs*9), p.(Gly329Glufs*3), and exon 3–4 deletion (p.Val75*). All of them are predicted to lead to NMD or cause significant protein truncations, clearly indicative of a loss‐of‐function. Additionally, we identified three novel missense variants and applied functional testing to assess catalytic function (Figure 4). None of the identified variants p.(Pro213Leu), p.(Arg99His), and p.(Arg384Trp) were in proximity to the active site. We tested each variant's activity in a cell‐based assay (Figure 4A). The expression of each C‐terminally‐3xFLAG tagged construct was analyzed by immunoblot to test for differences in protein stability (Figure 4B). Analysis of the sulfatase activity from wildtype ARSG transfected cells showed the expected robust activity increase by approximately fivefold compared to untransfected cells (Figure 4A). In contrast, neither lysates from the cells overexpressing the three tested ARSG mutants p.(Pro213Leu), p.(Arg384Trp), and p.(Arg99His) revealed increased total sulfatase activity above the background of untransfected cells (Figure 4A). Immunoblot analysis did not reveal any major differences in any of the constructs bearing missense variants compared to the wildtype ARSG protein, which was detected primarily as a major polypeptide slightly above 70 kDa. Minor additional polypeptides were detected, representing possibly SDS‐resistant dimers (~130 kDa) and proteolytic fragments. However, none of the missense variants affected protein stability (Figure 4B). In summary, our experiments confirmed the loss of function of each tested missense variant without affecting the protein stability.

## 4 DISCUSSION

In the past, biallelic variants in *ARSG* were tentatively assigned to cause “USH type IV” or atypical Usher syndrome.^2^ However, this classification was under discussion, and classification as a new type of “deafblindness” syndrome was suggested more recently.^1^, ^7^ It is clear that *ARSG*‐USH can be considered an (ultra‐)rare subtype, with only 22 affected subjects described thus far. We have now expanded the *ARSG* disease cohort to a total of 31 cases, suggesting that the number will likely further increase and that variants in *ARSG* as a cause for USH are underdiagnosed. This finding is also supported by a large‐scale study analyzing the carrier frequency and genetic prevalence of autosomal recessive IRD^21^ in which carrier frequencies between 0.00041 (Latino) and 0.0012 (Europe/non‐Finnish) were found for pathogenic *ARSG* variants. *ARSG* should, therefore, be included in standard‐of‐care genetic testing in subjects with typical retinal and HL symptoms (even without vestibular symptoms), or with an apparent isolated IRD. Of note, one of the subjects from this study presented the first symptoms (night blindness) already at 25 years, suggesting an earlier disease onset, as described. Another interesting finding is the large number of patients of Portuguese origin: 9 (7 subjects here) out of 31 patients are from Portugal or are of Portuguese origin.^10^ We identified the same novel *ARSG* variant c.1150C>T, p.(Arg384Trp) in 3 families of Portuguese descent (subjects 4–6, 13), highlighting a potential founder variant.

The initial finding that variants in *ARSG* cause USH but not a full‐blown LSD was rather unexpected, given the phenotype of the animal models and the critical role of ARSG in the lysosomal turnover of HS. Both *Arsg* knockout mice and ARSG‐deficient dogs show all signs of a mucopolysaccharidosis/Sanfilippo‐type LSD, including neurological symptoms (primarily manifested as ataxia) and storage in peripheral tissues. These findings are compatible with the function of ARSG in the lysosomal degradation of HS. The explanation for this apparent discrepancy between the animals and human subjects remains enigmatic. However, it should be noted that a similar situation exists for other genes typically causing LSDs, namely subtypes of NCL and mucopolysaccharidosis: variants in *CLN3*, *MFSD8* ^22^, ^23^ and *HGSNAT*.^24^ For these genes, the associated phenotypes can be categorized into two distinct types of autosomal recessive conditions: full‐blown early‐onset LSD with severe neurodegeneration,^25^, ^26^, ^27^ or isolated or nonsyndromic IRD such as RP.^22^, ^23^, ^24^, ^28^, ^29^ The effect of the underlying variants can likely explain these different phenotypic characteristics: while complete loss‐of‐function leads to the extreme LSD‐type, hypomorphic, or milder variants, resulting in residual function will often cause isolated IRD. The molecular function of MFSD8 is unknown, and residual functionality has not yet been proven on a functional level. The function of CLN3 in the clearance of glycerophosphodiesters from lysosomes has been discovered only recently,^30^ and the analysis of the functionality of patient mutations is still pending. Very recently, a very mild case with MPSIIIA (caused by *SGSH* variants) was described,^31^ mostly presenting clinical features of USH, including RP and SNHL, further strengthening the observation that these features might present the mildest forms of at least some LSD.

It could be speculated that a similar situation exists for *ARSG* whereby the variants that have been described so far are milder (hypomorphic) variants, which manifest with a relatively mild phenotype with HL and RP, similar to the situation observed in cases with isolated IRD due to variants NCL/mucopolysaccharidosis genes. Indeed, the assay used so far to test the functionality of the identified ARSG variants is not sensitive enough to reveal any minor residual activity, and an assay to monitor endogenous ARSG activity does not yet exist. However, two previously described ARSG variants (p.Asp44Asn and p.Asp45Tyr) affect amino acid residues in the active site,^2^, ^9^ suggesting a total loss‐of‐activity rather than a hypomorphic variant coding for an enzyme with residual enzymatic activity. Moreover, several frameshift variants have been identified, making it very unlikely that these variants represent hypomorphic variants with residual activity.

The identification of the p.(Arg99His) variant in a USH case is a second strong argument that hypomorphic variants alone cannot explain the difference between the experimental animal models and the human subjects identified so far; given that the same variant leads to full‐blown LSD in dogs, but mild USH phenotype here, indicating that other mechanisms underlie this discrepancy between the species.

Further work is needed to understand the function of ARSG in human USH subjects and the pathological cascades leading to disease manifestations. The association between the impaired degradation of HS and the primary cause of retina degeneration has not yet been clearly established. Furthermore, it is unclear if the affected cases show signs of LSD that are below the threshold needed to induce the clinical signs observed in mucopolysaccharidosis subjects, including typical neurological symptoms, organomegaly, and vacuolization in primary cells such as fibroblasts. It is notable that the first *ARSG* case with signs of ataxia was described in a recent case report,^12^ similar to the phenotype in the animal models. Mild cerebellar atrophy was observed in one of the subjects but did not manifest in ataxia. This feature should be closely examined in all identified cases and might only manifest with aging. Interestingly, ataxia develops late in *Arsg* knockout mice.^4^ Altogether, these findings suggest that other organs might be affected but only moderately without the manifestation of clinical signs.

A possible explanation for the milder manifestation in human subjects could be the abundance of the ARSG, 3‐*O* modified HS substrate. If less 3‐*O* HS is generated (e.g., due to lower expression of the responsible 3‐*O* sulfotransferases), this would lead to a lower storage burden and represent a vital disease modifier. Alternatively, compensatory pathways in the degradation of HS in general or, more particular, the desulfation of 3‐*O* sulfate, for example, by another sulfatase in humans, could also explain the obvious differences. Additional biochemical studies are necessary to better understand these features of USH type IV and functional studies on patient‐derived material (e.g., skin biopsies), which could also indicate presymptomatic signs of lysosomal storage.

Our study greatly expands the genetic landscape of *ARSG*‐USH IV with novel pathogenic variants, confirming previously found variants and expanding the number of known affected subjects by ~30%. The small number of subjects, however, may limit the generalizability. Our findings highlight that USH IV likely has been significantly underdiagnosed and emphasize the need to test molecularly unresolved subjects with USH. As HL is a late‐onset feature in our cases, testing for *ARSG* variants should also be considered for patients with apparent isolated IRD.

## AUTHOR CONTRIBUTIONS

All authors contributed to the study's conception and design. Material preparation, data collection, and analysis were performed by M.B; V.DM.; S.VdS.; M.VH.; I.A.; I.B.; W.Z.; M.DB; V.S.; S.H.; S.V.; E.L.; J.J.; I.V.; J.D.; C.A.; C.C.; A.A.; R.H.; AL.C.; JP.M.; C.Z.; E.DB; M.D. The first draft of the manuscript was written by M.D., and all authors commented and approved the final manuscript.

## FUNDING INFORMATION

This research was funded by Fondation Voir et Entendre (CZ), LABEX LIFESENSES (ANR‐10‐LABX‐65) (IA/CZ), by ANR‐11‐IDEX‐0004‐0, IHU FOReSIGHT, ANR‐18‐IAHU‐0001 (IA/CZ), ANR‐18‐IAHU‐01 (FDB), by Retina France (IA/CZ), by Foundation Fighting Blindness center grant [C‐CMM‐0907‐0428‐INSERM04] (IA/CZ), BR‐GE‐0619‐0761‐INSERM (IA/CZ), UNADEV (IA/CZ) in partnership with ITMO NNP/AVIESAN, by the National Eye Institute, National Institutes of Health (WMZ, RBH), by Ghent University (BOF20/GOA/023) (EDB) and EJPRD19‐234 Solve‐RET (EDB), by FWO‐1802220N (EDB).

## CONFLICT OF INTEREST STATEMENT

None of the authors declares any competing interests or conflicts of interest.

## Supporting information


**Table S1.** Supporting Information.

**Figures S1‐S4.** Supporting Information.

**Data S1.** Supporting Information.

## Data Availability

All data relevant to the study are included in the article or supplementary information. Data not included are available upon reasonable request (mdamme@biochem.uni-kiel.de/elfride.debaere@ugent.be).
