# Supplementary material for: Expanding the genetic landscape of Usher syndrome type IV caused by pathogenic ARSG variants
Source: Clin Genet. 2024 Aug 28;107(1):44–55. doi: 10.1111/cge.14614 (PMC11608847; doi:10.1111/cge.14614)
Supplement: Supplementary file 2 — Figures S1‐S4. Supporting Information. [file CGE-107-44-s001.pdf]

Supplemental Figure 1

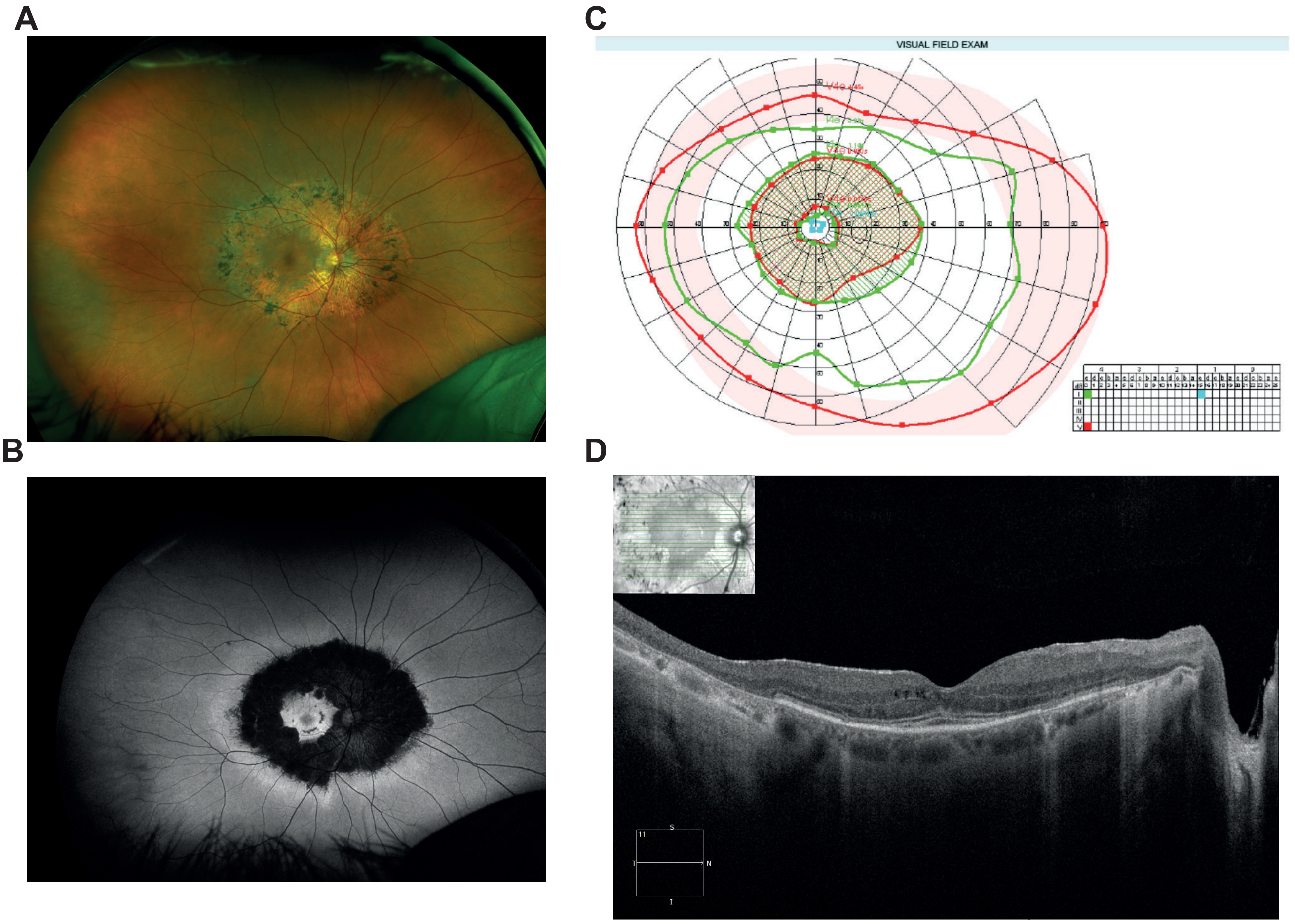

Supplemental Figure 1. Clinical imaging of subject 3. (A) Right eye Optos CFP and **(B)** FAF images, **(C)** kinetic visual field (MonCvONE, MetroVision), and **(D)** OCT raster scan through the fovea (Cirrus HD).

Supplemental Figure 2

A Left eye Right eye

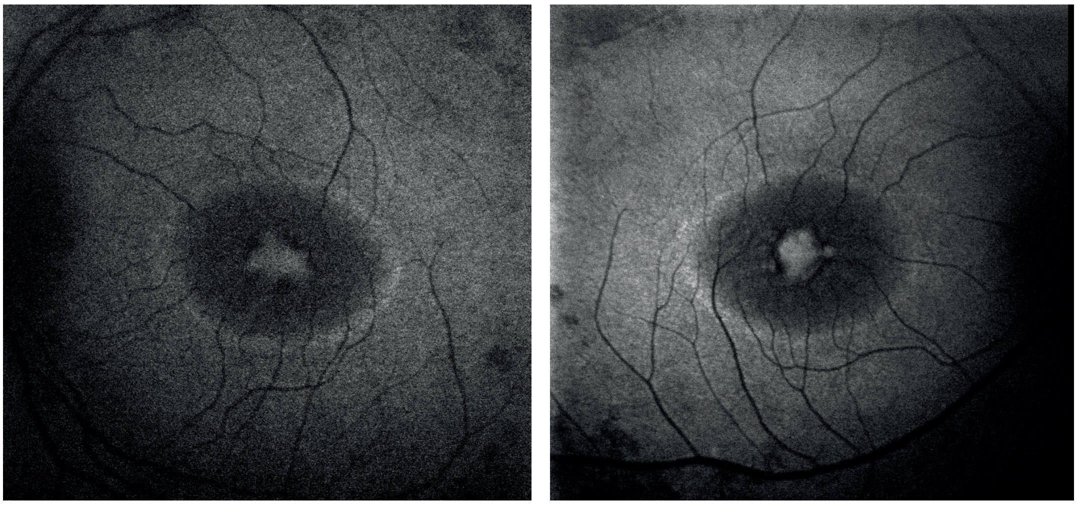

B

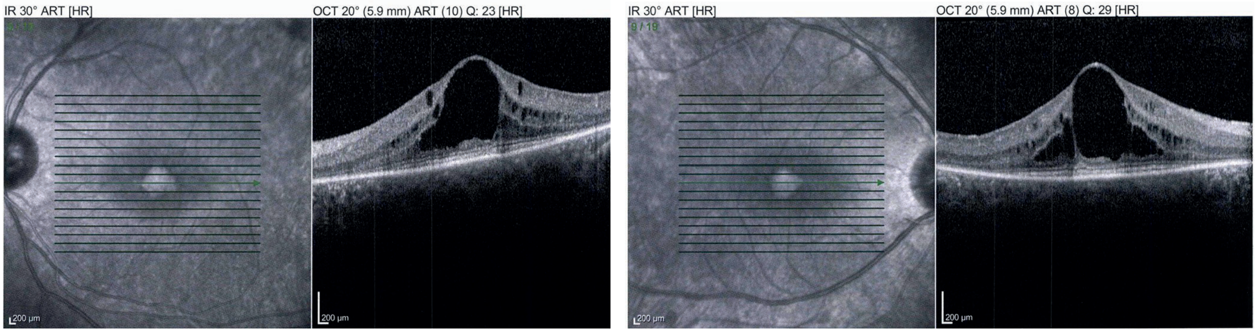

C

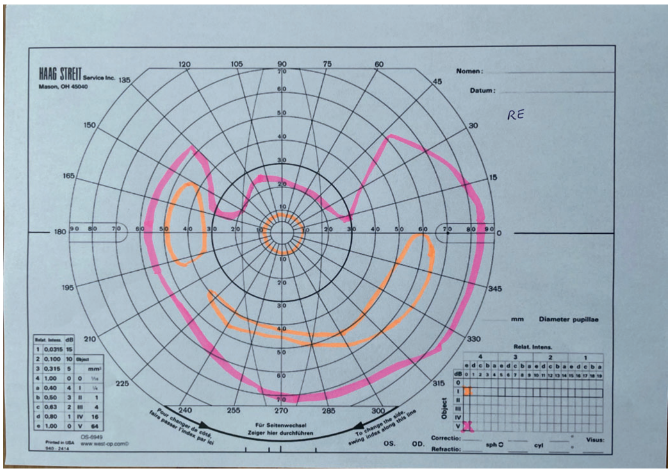

Supplemental Figure 2. Fundus autofluorescence (FAF), and optical coherence tomography (OCT) of the right and left eyes of subject 2 and visual field. (A) FAF showed a ring of parafoveal hyper-autofluorescence, a central spot of hyper-autofluorescence, and more peripherical hypo-fluorescence changes **(B)**. Spectral-domain OCT showed preserved outer retinal layers at the macula with cystoid macular oedema in each eye **(C)** The visual fields showed a peripheral reduction more pronounced in the upper part in each eye

Supplemental Figure 3

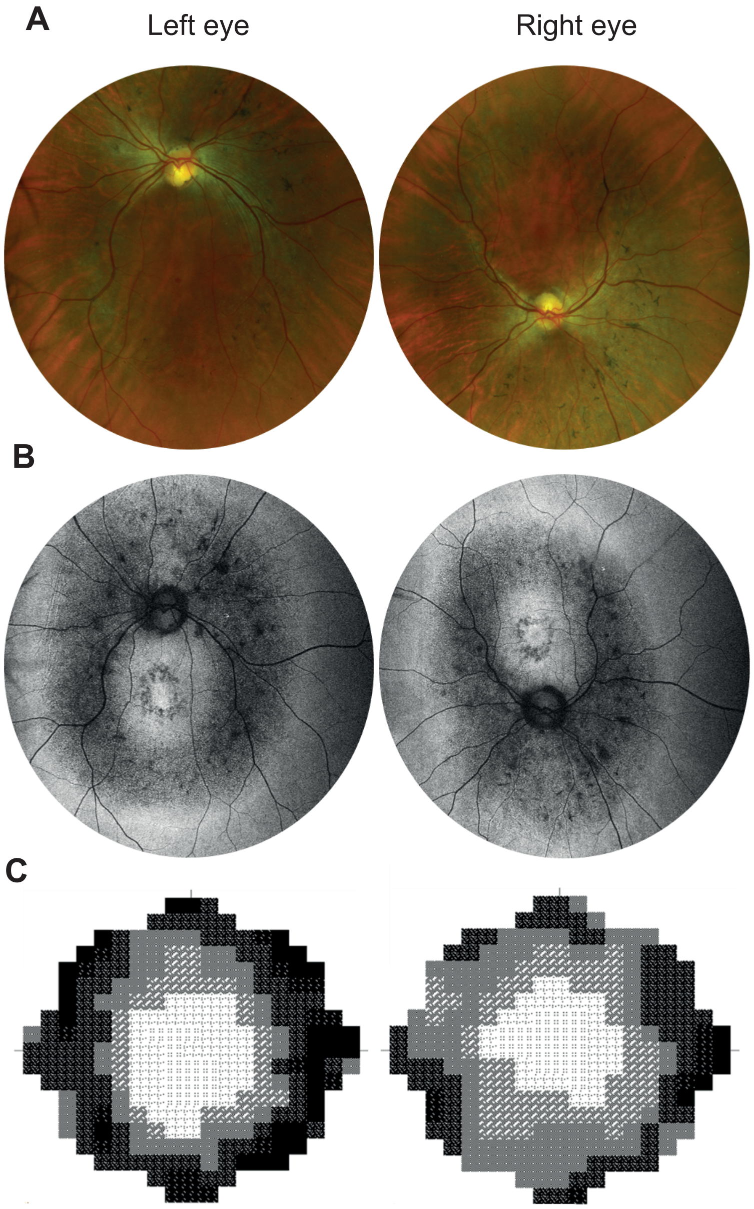

Supplemental Figure 3. Ultra-widefield (UWF) color fundus photography, ultra-widefield fundus autofluorescence, and Humphrey field analyzer (HFA) 10-2 of the right and left eyes of subject 12. (A) Patches of outer retinal atrophy and bone spicule hyperpigmentation are observed along the vascular arcades **(A)** and appear hypoautofluorescent in UWF fundus autofluorescence **(B)**. Perifoveal atrophy can be identified as hypoautofluorescence patches encircling the fovea. On HFA **(C)**, severe constriction is observed with preservation of retinal sensitivity limited to the central 5º.

Supplemental Figure 4

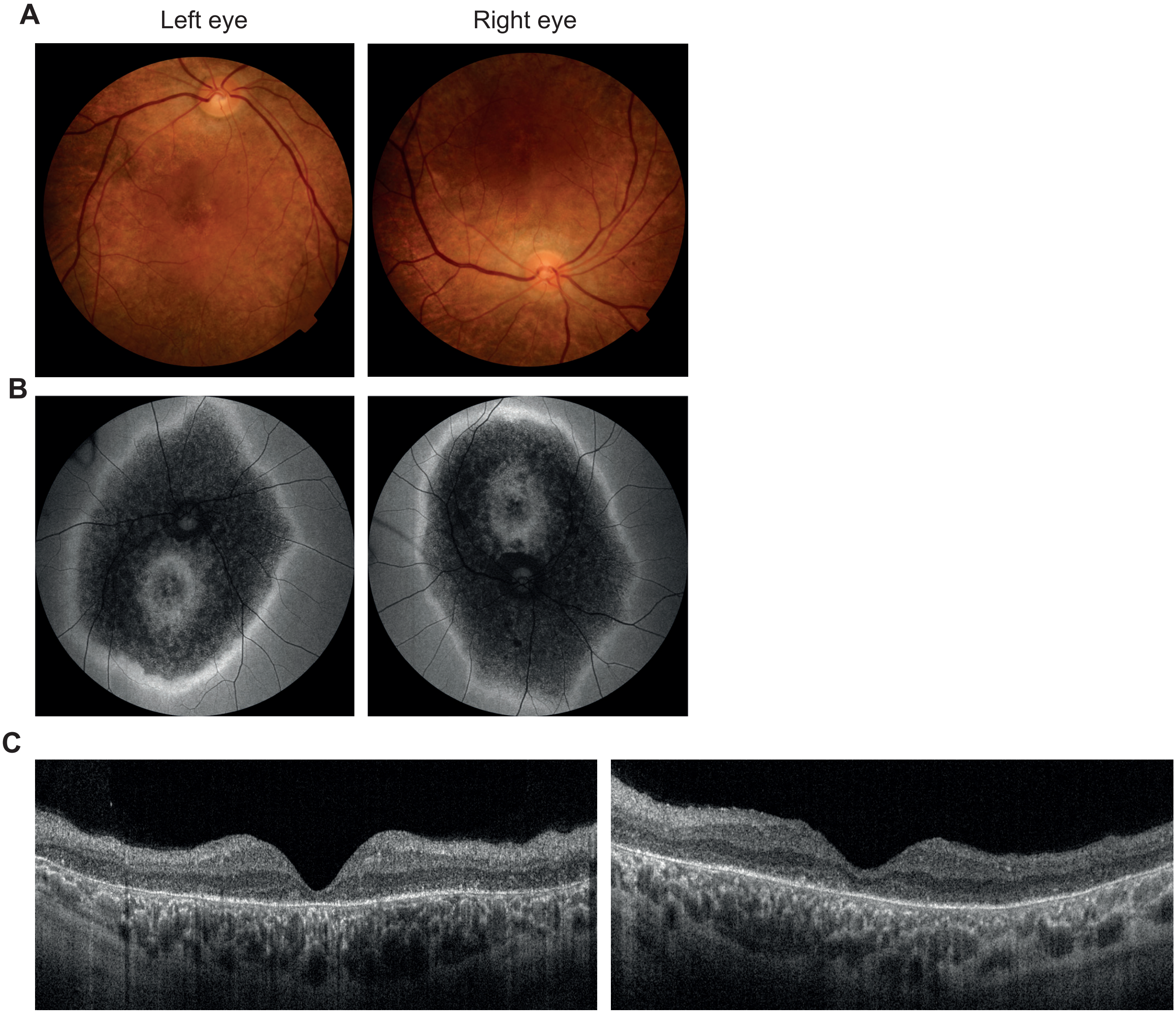

Supplemental Figure 4. Color fundus photography (CFP), blue light fundus autofluorescence (FAF), and spectral domain optical coherence tomography (SD-OCT) of the right and left eyes of subject 13. (A) CFP depicts patches of outer retinal atrophy along the vascular arcades and in the macular area, involving the fovea. These areas appear hypoautofluorescent on FAF **(B)**. A hyperautofluorescent ring can be seen delimitating the atrophic central retina and the presumably healthy peripheral retina. On SD-OCT **(C)**, subfoveal atrophy is shown, with loss of integrity of the outer retinal layers and the retinal pigment epithelium.
