# Supplementary material for: Expanding the genetic landscape of Usher syndrome type IV caused by pathogenic ARSG variants
Source: Clin Genet. 2024 Aug 28;107(1):44–55. doi: 10.1111/cge.14614 (PMC11608847; doi:10.1111/cge.14614)
Supplement: Supplementary file 3 — Data S1. Supporting Information. [file CGE-107-44-s002.docx]

**Supplementary Information**

**Supplemental Methods**

***Clinical examinations at different centers:***

**University Hospital Leuven, Leuven, Belgium.**

The patient was assessed first by a resident and afterwards by an ophthalmologist specialized in inherited retinal diseases. Best-corrected visual acuity was measured using ETDRS letters. Amsler grid, Snellen reading chart, and color vision by Ishihara test plates were assessed. Slit-lamp examination of the anterior segment and fundoscopy were performed both before and after mydriatic drops. Technical examinations and multimodal imaging included spectral-domain optical coherence tomography (SD-OCT) (Carl Zeiss Meditec - Germany), fundus autofluorescence (FAF) (Heidelberg Engineering, Heidelberg, Germany), central visual field testing by Octopus-900 semi-automated kinetic perimetry (Isopter V4) (Haag-streit, Swiss), peripheral visual field on a manual Goldmann perimeter and ultra-widefield color fundus photography (Clarus - Carl Zeiss Meditec - Germany).

**Ophthalmic Genetics and Visual Function Branch, National Eye Institute, National Institutes of Health, Bethesda, USA.**

An assessment of visual function and retinal health was completed with a specialist in inherited retinal diseases. This included measurement of best-corrected visual acuity using an ETDRS chart, color vision testing (Farnsworth D15), slit-lamp assessment of the anterior segment, and exam of the ocular fundus. Kinetic perimetry was obtained on the MonCvONE perimeter (Metrovision – Perenchies, France). Color fundus photography (CFP) and fundus autofluorescence images were performed with an ultra-widefield retinal imaging device (Optos - Dunfermline, Scotland). Optical coherence tomography (raster and macular cube scans) was obtained on a Cirrus HD (Carl Zeiss Meditec – Dublin, CA, USA). In addition, ancillary testing included MP1 microperimetry (Nidek Technologies – Padua, Italy) and electroretinography (LKC Technologies - Gaithersburg, MD, USA).

**Reference Center for Rare Diseases RefeRet at Centre Hospitalier National d'Ophtalmologie des Quinze-Vingts, Paris, France.**

Family members available for the study underwent full ophthalmic examination with assessment of best-corrected visual acuity, kinetic and static perimetry, and color vision using 15 desaturated hues. Full-field and multifocal electroretinography (ERG) was performed using Dawson, Trick, Litzkow (DTL) recording electrodes and incorporated the International Society for Clinical Electrophysiology of Vision standards (Espion e2 system and ColorDome Ganzfield stimulator (Diagnosys UK Ltd, Cambridge, England) for full-field ERG and Veris II system (Electro Diagnostic Imaging Inc, San Francisco, California) for multifocal ERG). The clinical assessment was completed with fundus autofluorescence imaging and OCT (with the use of HRAII and Spectralis OCT, respectively; Heidelberg Engineering, Dossenheim, Germany).

**Centro Hospitalar e Universitário de Coimbra (CHUC), Coimbra, Portugal.**

All patients enrolled in the study underwent a comprehensive ophthalmologic examination by an IRD specialist (JPM) comprising best-corrected visual acuity (ETDRS letters), dilated slit-lamp anterior segment, and fundus biomicroscopy. Functional testing and multimodal imaging included 10-2 Humphrey field analyzer (HFA) (Zeiss 750i, Carl Zeiss, Germany), standard 45°-field color fundus photography (CFP) (Nikon Digital SLR Camera D7000, Nikon Corporation, Japan), blue light fundus autofluorescence (FAF) (HRAII, Heidelberg Engineering, Heidelberg, Germany) ultrawidefield (UWF) CFP and UWF-FAF imaging (Optos California, Optos GmbH, Germany), and spectral-domain optical coherence tomography (SD-OCT) (Spectralis, Heidelberg Engineering, Heidelberg, Germany).
